# Healthcare graduate students' perceived control and preventive behavior for COVID-19 in Japan and the United States: A cross-sectional study

**DOI:** 10.3389/fpubh.2022.965897

**Published:** 2022-10-27

**Authors:** Renaguli Abuliezi, Akiko Kondo, Kosuke Niitsu, Erika Ota

**Affiliations:** ^1^Graduate School of Health Care Sciences, Tokyo Medical and Dental University, Tokyo, Japan; ^2^School of Nursing and Health Studies, University of Washington Bothell, Bothell, WA, United States; ^3^Department of Global Health Nursing, St. Lukes International University, Tokyo, Japan; ^4^The Tokyo Foundation for Policy Research, Tokyo, Japan

**Keywords:** COVID-19, perceived control, preventive behavior, individualistic and collectivistic culture, healthcare students, graduate students, perceived health competence, control model

## Abstract

**Background:**

Both individual and policy level perceived control are known to be positively related to preventive behavior, and both may differ among healthcare graduate students with different cultural backgrounds. This study compared the preventive health behavior and perceived control among domestic and international healthcare graduate students in Japan and the United States during the COVID-19 pandemic, and analyzed factors associated with preventive health behavior and perceived control.

**Methods:**

The study used a self-administered online survey, conducted at two universities in Japan and one university in the United States. The survey included sociodemographic data and scales of preventive health behaviors, perceived control (policy level), and perceived health competence (individual level). Association among variables were analyzed using structural equation modeling.

**Results:**

A total of 610 students (485 domestic and 125 international) in Japan and 231 students (220 domestic and 11 international) in the United States completed the survey. Participants' average age was 31.3 years, and 67.0% were female. Model fit of structural equation modeling was good (χ^2^ = 9.419, *P* = 0.151, comparative fit index = 0.995, RMSEA = 0.026). Japanese students had better preventive health behavior than American (β = −0.407, *P* < 0.001) and international students in both countries (β = −0.112, *P* < 0.001). However, Japanese students had significantly lower perceived control than American students (β = 0.346, *P* < 0.001) and international students in both countries (β = 0.188, *P* < 0.001). Overall higher perceived control (β = 0.175, *P* < 0.001) and being female (β = 0.141, *P* < 0.001) were significantly associated with better preventive behavior. Although higher perceived control was related to higher perceived health competence (β = 0.295, *P* < 0.001), perceived health competence was not associated with preventive behavior (β = 0.025, *P* = 0.470). Religion was not associated with perceived control or preventive behavior.

**Conclusion:**

Nationality was identified as the main factor associated with both perceived control and preventive behavior. Policy level perceived control was more strongly associated with preventive health behavior than individual level perceived health competence. Further investigations in the contribution of specific cultural dimensions associated with perceived control and preventive behaviors are recommended.

## Introduction

The COVID-19 pandemic has been causing a healthcare crisis, worldwide, since late 2019. Globally, there have been more than 500 million confirmed cases of COVID-19, including 62,94,969 deaths as of June 2022 (1). The number of infections due to the new variants of the SARS-CoV-2 virus continue to rise even after the availability of the vaccine (2). The Centers for Disease Control and Prevention identifies infection prevention and individual behavior as critical measures for the containment of the pandemic (3).

Healthcare workers face a greater risk of COVID-19 infection than the general public because of frequent exposure to infected patients (4). Healthcare students have also become a high-risk infection group because of their volunteering to take on clinical roles in healthcare facilities to deal with patient overload during the pandemic, in addition to their studies (5–8). A systematic review conducted on the COVID-19 related knowledge, attitudes, and prevention practices among university students worldwide, demonstrated that non-medical students have better preventive practices against COVID-19 than medical students, although the latter tend to have more knowledge and awareness (9). Studies on preventive behavior during the COVID-19 pandemic among healthcare students identified associated factors, such as sociodemographic characteristics, current working states, knowledge, and risk perception against COVID-19 (10–14). In another study, differences were observed in preventive behavior practice among students of different nationalities whereby Chinese university students showed higher preventive practice than Japanese and Korean students did (15). In addition, we reported that perceived control was also positively associated with better preventive behavior among Japanese undergraduate nursing students during the pandemic (16).

Perceived control is referred to as a person's subjective beliefs on the capability he or she possesses to influence both one's own internal status and external environment or outcome (17–20). Perceived control has a positive association with better performance, coping with stress, and success in behavior changes (21). High self-efficacy and high perceived behavioral control were associated with improved health behaviors among young adults (22–24). In Hornsey et al. (25) large cross-national study that compared perceived control among 38 nations, Japan showed the lowest perceived control while the United States showed a moderate level of perceived control. Robinson and Lachman (26) summarized, that the factors related to high perceived control were: being male, increased age, and high socioeconomic status; race and culture were also related to perceived control. Morling et al. (27) showed that Japanese and American cultures had an opposite effect on coping with the surrounding environment, with the latter tending to influence the surroundings and the former tending to adjust. Moreover, religion played a role in the development of culture through traditions. A review summarized that religion influenced self-control and resulted in influencing health and wellbeing (28). Previous studies demonstrate that perceived behavioral control impacts engaging and adapting preventive behavior among general university students in Bangladesh (29) and Chili (30). However, in these studies, most of the scales employed measured perceived control at the individual level, or internal control. Meanwhile, these studies only focused on general undergraduate students and did not include graduate students as participants.

Graduate students are confronted with numerous academic and life challenges because of their conflicting roles as adult students and other roles in their life (e.g., wife/husband, parent); some graduate students also need to work full-time while pursuing their academic degrees (31). The pandemic may have brought even harder challenges for graduate students in healthcare studies, by adding to their responsibilities in clinical settings, apart from their studies. International graduate students with diverse cultural backgrounds may face additional challenges during the pandemic in the host countries, such as staying apart from their families, financial constraints, and educational stress (32–34). We reported that more policy level or external environment perceived control also had a positive association with better preventive behavior among Japanese undergraduate nursing students during the pandemic (16). In another study, we reported the factors related to mental health among both undergraduate and graduate nursing students in Japan and the United States; the results indicated that perceived control, not only at the individual level but also at the policy level, can buffer the mental health effects caused by preventive behavior during the COVID-19 pandemic. However, this study excluded international students due to the small number of international students in the nursing department (35). Therefore, we further collected data from medical and dental graduate students. To the best of our knowledge, no studies conducted on factors related to the perceived control have been reported among healthcare graduate students, including international students with different nationalities. Moreover, the link between policy level perceived control and preventive behavior during the COVID-19 pandemic among them is yet to be elucidated.

Investigating the differences in the preventive behaviors and perceived control among graduate students from different cultural backgrounds is necessary to provide targeted and culturally sensitive care to improve their health outcomes, assist in achieving success during their graduate programs, and establish a foundation for present and future healthy behaviors.

The current study was aimed: (1) to compare perceived control and preventive health behaviors among domestic and international healthcare graduate students in Japan and the United States; (2) to determine the factors related to preventive health behavior; and (3) to determine the factors associated with perceived control during the pandemic from a cross-cultural perspective. We proposed the following hypotheses:

Hypothesis 1: Levels of perceived control and preventive health behavior differ among student groups; Japanese healthcare graduate students have the lowest perceived control.
Hypothesis 2: Perceived control, nationality, and religion are associated with preventive behavior.
Hypothesis 3: Nationality and religion are the main predictors of the level of perceived control during the pandemic.

## Materials and methods

### Conceptual theoretical framework

**Figure 1:**
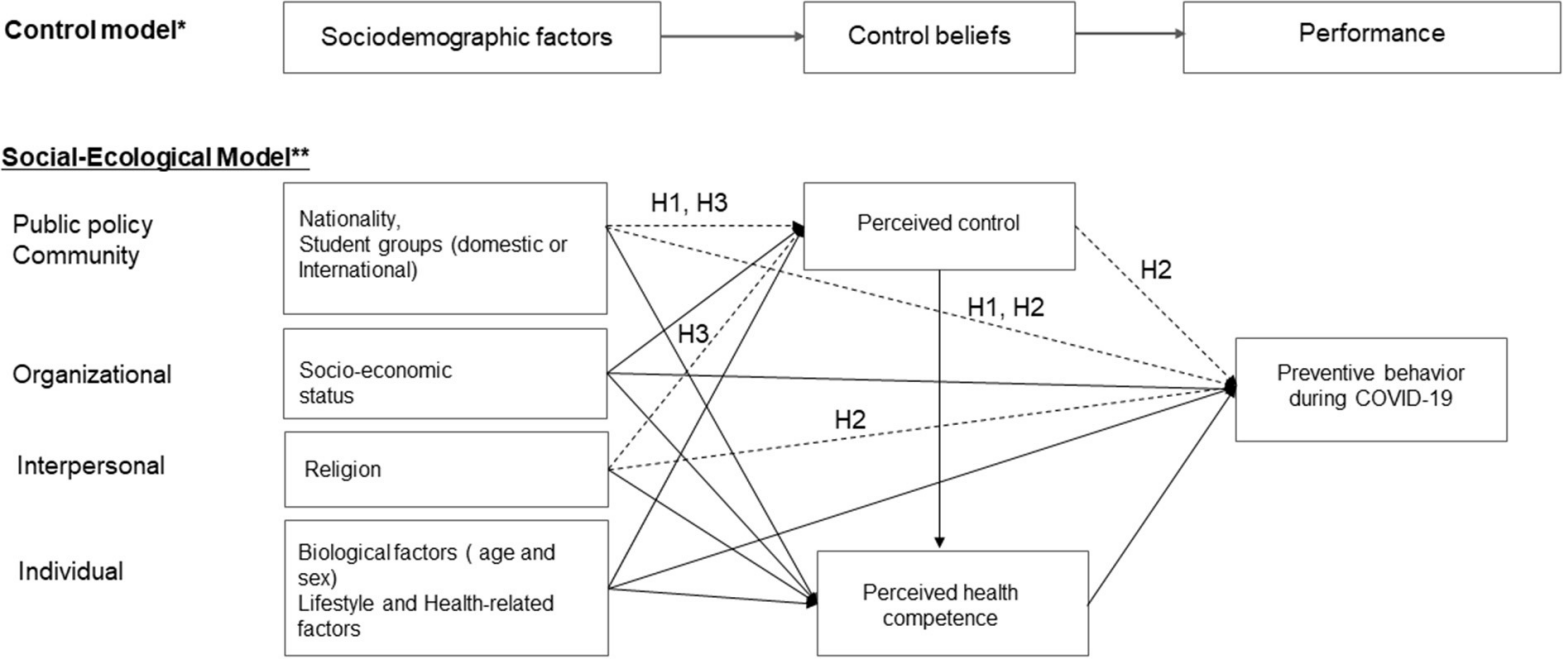
Conceptual framework. Source: *Adapted from (26). **4. Important Theories and their Key Constructs (Figure 4) In Social and Behavioral Theories, e-Source Behavioral Social Science Research, available at https://obssr.od.nih.gov/wp-content/uploads/2016/05/Social-and-Behavioral-Theories.pdf (Retrieved August 29, 2022).

This study's conceptual theoretical framework (Figure 1) utilized the Control Model, which is adapted from Robinson and Lachman (26). and Socio-ecological model, which is adapted from National Institute of Health (32). The Control Model suggests that control beliefs are affected by sociodemographic factors that affect the behavioral skills, which in turn affect the performance and outcome of a person (P217) (26). The sociodemographic factors and control beliefs were also classified into five levels in the Socio-ecological model: individual, interpersonal, organizational, community, and public policy factors, which emphasize the idea that behaviors not only shape but are also shaped by the social environment (36).

In the current study, biological factors (37) and lifestyle item, which were considered to be related to general health in previous studies, were also added in sociodemographic factors and included in individual levels of Socioecological model. Religion was included in the interpersonal level, socio-economic status was included in organizational or interpersonal level, nationality and student status (domestic or international) were included in community and policy levels. Control beliefs include concepts of perceived control and perceived health competence. Individuals who believe that they have influence over policy decisions in their communities and that they have strong control over their own behavior are likely to experience more positive health outcomes (38). Because preventive measures are closely related to government policies and strategies, students who are aware of these policies or are often politically concerned may have higher preventive behavior engagements based on public policy (16). Perceived health competence is a measure of self-efficacy in terms of capability of managing own health outcomes (39). Perceived control measures the control beliefs at more community and policy levels as indicated in upper level in the Figure 1, while the perceived health competence measures it at individual levels as indicated in the lower position in the Figure 1. Preventive health behaviors refer to an individuals' performance regarding preventive measures.

### Study design and participants

The current cross-sectional study was conducted at two universities: Tokyo Medical and Dental University and St. Luke's International University in the Tokyo metropolitan area in Japan, and University of Washington, located in the northwestern part of the United States. Study participants included both international and domestic students (above 18 years of age) who were enrolled in graduate programs (Masters and Doctoral) in healthcare areas during the research period, who agreed to participate. These universities were selected due to the researchers' accessibility (convenience sample).

### Data collection

Data collection was achieved by using a self-administered online survey *via* Google Forms. We emailed an invitation to all eligible participants with a link to the survey. Since two selected universities in Japan were medical universities, emails were sent to all graduate students enrolled in these two universities in healthcare related majors (medical, dental, public health, and nursing programs). We also emailed to the University of Washington's graduate students registered in different nursing programs across the three campuses, and dental students at the Seattle Campus, where research collaborations were available. Data were collected from January to May in 2021, in both countries.

### Ethical considerations

Ethical approval was obtained from Institutional Review Boards of all three universities for this study (see Ethics Statement, for approval number). Participants were invited to participate in this study voluntarily and were informed that their confidentiality and anonymity were protected. An Amazon gift card (worth $4.5 for participants from both Japanese universities and $10 for participants from University of Washington) was given to the participants who provided an email address after completing the survey to compensate for their time and effort. A different Google form, which did not have any links to the survey, was used to collect the email addresses.

### Measurements

The questionnaire comprised four sections: preventive behavior, perceived control, perceived health competence, and sociodemographic data. The first section was created by the authors, which included 17 questions (See Supplementary Table 1) regarding the frequency of preventive health behavior engagement during the COVID-19 pandemic (e.g., avoidance of crowded, closed, and close-contact settings; wearing a mask in public, etc.). The frequency of preventive behavior engagements was measured using a 4-point Likert scale with options of never = 0, sometimes = 1, often = 2, and always = 3; a higher score indicated more frequent preventive behavior engagements. The questionnaire items were initially created by the first two authors based on previous studies (40). Thereafter, a pilot study was conducted with 11 graduate students and revised to the final form through multiple discussions involving all the authors. A good reliability was confirmed for preventive health behaviors with Cronbach alpha scores of 0.792 for students in Japan and 0.839 for those in the United States.

The second section of the questionnaire assessed the perceived control by using the Control and Self-Efficacy (CASE) Scale (38). CASE is a 10-item 5-point Likert scale, adapted from the British General Household Survey (GHS) Social Capital scale, and includes the perceived influence on both individual and societal levels (41). CASE includes three domains. Domain 1 measures control over community affairs. Domain 2 measures influence over political decisions, which can measure perceived control at one's external environment, outcome, or policy level. Domain 3 measures control over personal life, which can measure perceived control at one's internal status or individual level. This scale uses 5 points ranging from strongly disagree = 1 to strongly agree = 5, and a higher total score indicates a higher level of perceived control and self-efficacy. The authors translated it into Japanese, confirmed its reliability (Cronbach alpha = 0.706) and validity with Japanese participants (16). In the current study, the Cronbach alpha scores of perceived control scales were 0.699 and 0.682 for students in Japan and the United States, respectively. Permission to translate and use the scale was obtained from the creator, Salehi (38).

The third section of the survey measured perceived health competence using the Perceived Health Competence Scale (PHCS) (39). This scale measures the degree of participants' feeling regarding the effectiveness of self-health outcome management using an 8-item scale that also uses a 5-point Likert format from strongly disagree = 1 to strongly agree = 5, with a higher total score indicating a higher health competence. The reliability and validity of the Japanese-translated version of the scale have been confirmed (42). In the current study, the Cronbach alpha scores of perceived control scales were 0.869 and 0.864 for students in Japan and the United States, respectively. Permissions were obtained to use these scales from the developers and translators.

The last part of the survey was on the sociodemographic factors, which included nationality, student groups (domestic or international), socio-economic status, religion (Buddhism, Shintoism, Christianity, Islam, Hinduism, and no religious beliefs), biological factors (age and sex), and lifestyle and health related items (alcohol drinking, sleeping hours, and having chronic conditions requiring regular checkups) (39, 43–45). Socio-economic status included living conditions (living with somebody or not), current work status (working full-time, part-time, or not working), and having medical-related license.

### Analysis

The Shapiro-Wilk test was used for examining the data normality; basically, non-parametric method was used for data analysis since all the continuous variables (e.g., age, sleeping hours, total scores of preventive behavior, perceived control, and perceived health competence) were not normally distributed.

Four student groups were collapsed into three: Japanese domestic, American domestic, and international students (from both countries) because of the limited number of international students in the United States. Since international students between countries did not show significant differences in the total scores of the three scales, and medians of preventive behavior scores of international students in both countries were closer than other domestic student groups (see Supplementary Table 2), international students in both countries were categorized into one group.

Chi-square test or Fisher's exact test was used to compare nominal variables such as sex and religious beliefs among three student groups. Since the number of some of the religious beliefs in our sample was quite small (e.g., Shintoism = 14, Hinduism = 7), for further analysis, we used the variable of “have religious beliefs or not” to analyze for association with other variables. Kruskal–Wallis test was used to compare continuous variables among three student groups. Mann-Whitney *U*-test was used to compare continuous variables between each independent pair of groups. Bonferroni correction was applied for multiple comparison according to the number of analysis (e.g., 0.05/3 = 0.017).

Factors associated with preventive health behaviors, perceived control, and perceived health competence were initially analyzed for each student group (Japanese domestic, American domestic, and International Students) in bivariate analyzes. Variables that were significant (*P* < 0.05), at least in one student group, were selected to be entered into each multivariate binary logistic regression, to determine the related factors after controlling for other variables. In bivariate analysis, factors that were inversely correlated with the three outcome variables (preventive behavior, perceived control, and perceived wellbeing) across student groups were considered to have interaction effects with the student group variable. Thereafter, the potential interaction variables with the student groups were entered into the logistic regression analysis as interaction terms.

Preventive health behavior (Median = 40.0, interquartile range: IQR = 36.0–44.0), perceived control (Median = 33.0, IQR = 29.0–36.0), and perceived health competence (Median = 29.0, IQR = 24.0–32.0) were sorted into two categories (less than median scores = 0, equal and greater than median scores = 1) as a dependent variable in multivariate binary logistic regression. The normality of residuals was confirmed by the Durbin-Watson ratio (between 1.5 and 2.5) (46). Multicollinearity was checked using the variance inflation factor (VIF) (<10.0), and model fit was checked by Hosmer-Lemeshow test (>0.05). An alpha level of 0.05 was set to be significant in all logistic regressions.

Since preventive behavior and perceived control showed significant differences in different student groups (Japanese, International, and the American), further logistic regression analysis was performed to explore if the following five nationality categories were related to preventive behavior and perceived control. In the second logistic regression, students were categorized into five groups based on their nationality or regions: Japan (*n* = 477), Mainland China (*n* = 49), the United States (*n* = 201), other Asian countries or regions (*n* = 69), and non-Asian countries (excluding the United States) (*n* = 26). Except for the first three groups which are distinguished by a single country, the other two groups included multiple countries or regions based on whether the geographical location of the country or region is in Asia.

Structural equation modeling (SEM) was used to test for the relationships among variables described in our theoretical framework. Variables that were significant (*P* < 0.05) in logistic regressions were selected for SEM. In logistic regression, although religion was not associated with perceived control or preventive behavior and perceived health competence was not associated with preventive behavior, these variables were included in the SEM analysis since the former was the main predictor, and the latter was a confounder of the association between perceived control and preventive behavior in the current study hypothesis. Model fit was reported with a chi-square test (*P* > 0.05), comparative fit index (>0.97), and root mean square error of approximation (RMSEA) (<0.05). AMOS automatically estimated means and intercepts for missing values (47–50).

In SEM, three outcome variables, total scores of preventive behavior, perceived control, and perceived health competence, were all used as continuous variables because their model fit index were all better than the models with binary outcomes. Outliers were checked in the scatter plots with preventive behavior and perceived control. When two outliers were excluded, we got similar results; therefore, the two outliers were included in the final models. All the data were analyzed using Statistical Package for Social Sciences (SPSS) version 27 and IBM SPSS AMOS28.

### Power analysis

After data collection, *post hoc* power analysis (Mann-Whitney *U*-test with alpha level = 0.017, power = 0.8, two tails) was carried out using G-Power 3.1.9.7 for each comparison student group. When Japanese domestic students (*n* = 485) and international students (*n* = 136) (allocation ratio = 3.6) were compared, effected size d = 0.33 was detected. Between international (*n* = 136) and American students (*n* = 220) (allocation ratio = 1.6), effected size d = 0.37 was detected; between Japanese (*n* = 485) and American students (*n* = 220) (allocation ratio = 2.2) effected size d = 0.27 was detected.

## Results

### Characteristics of participants

A total of 1,780 healthcare graduate students in Japan and 719 healthcare graduate students in the United States were invited for the survey. A total of 610 (485 domestic and 125 international) students in Japan and 231 (220 domestic and 11 international) students in the United States participated in this study with response rates of 34.3% and 32.1%, respectively. The mean age for all participants was 31.3 ± 7.9 years; 67% were female.

Sociodemographic characteristics of three student groups (Japanese, American, and International) are compared in Table 1. Japanese students were significantly older than American (*P* < 0.001) and international students (*P* = 0.025). More than half of the participants were female among all student groups. For religious beliefs, <30% of Japanese students and more than half of the students from the other two student groups were religious; over 40% of American students were affiliated with Christianity. Japanese students had shorter sleeping hours compared to the other two student groups (*P* < 0.001). International students in both countries were more likely to be living alone compared to the other two student groups (*P* < 0.001). Domestic students in both countries were more likely to work full-time or part-time compared to international students in both countries. Majority of the participants had a medical-related license: 40.8% were nurses, followed by dentists (21.3%), medical doctors (13.8%), laboratory technicists (5.3%), and pharmacists (1.5%), with an overall 7.12 ± 7.1 years of average working experience. In Japan, 39% of the international students were from Mainland China, 43.2% were from other Asian countries or regions (including Thailand, Vietnam, Taiwan, India, Indonesia, etc.,) and the remaining 17.8% were from non-Asian countries (e.g., Ghana, Germany, Tunisia, Brazil etc.,) (see Supplementary Table 3 for more details). In the United States, 94.4% of domestic students were American; among the remaining students, eight were from other Asian countries or regions (e.g., South Korea, Philippine, and Taiwan), two from Canada, and one student each from Ukraine and Mainland China. As for international students, five were from India, three from Taiwan, two from South Korea, and one each from Japan and Canada. There were no students from the United States among the international students in Japan, but there was one student from Japan among the international students in the United States.

**Table 1:** Comparison of sociodemographic characteristics and lifestyle among Japanese and the USA graduate students.

	**Japanese** **(*N* = 485)** **group 1**	***n***	**American** **(*N* = 220)** **group 2**	***n***	**International in** **Japan and** **the U.S** **(*N* = 136)** **group 3**	***n***	***P*** **-value**			
							**All groups**	**1 vs. 2**	**1 vs. 3**	**2 vs. 3**
**Sex (%)**										
Male	166 (34.8)	477	45 (20.9)	215	61 (46.6)	131	< 0.001^a^^,^^†^	< 0.001^a^^,^^†^	0.014^a^^,^^†^	< 0.001^a^^,^^†^
Female	311 (65.2)		170 (79.1)		70 (53.4)					
Age (years), median (IQR)	31.0 (26.0–38.0)	471	27.0 (24.0–32.0)	217	29.0					
(27.0–32.0)	133	< 0.001^b^^,^^†^	< 0.001^c^^,^^*^	0.025^c^	< 0.001^c^^,^^*^					
**Nationality categorize (%)**										
Japan	476 (99.2)	480	0 (0.0)	213	1 (0.8)	129	< 0.001^a^^,^^†^	< 0.001^d^^,^^*^	< 0.001^d^^,^^*^	< 0.001^d^^,^^*^
Mainland China	2 (0.4)		1 (0.5)		46 (35.6)					
USA	0 (0.0)		201 (94.4)		0 (0.0)					
Other Asian countries or regions	2 (0.4)		8 (3.8)		59 (45.7)					
Non-Asian countries (excluding USA)	0 (0.0)		3 (1.4)		23 (17.8)					
**Religious beliefs (%)**										
Buddhism	93 (19.9)	467	10 (5.0)	201	23 (17.8)	129	< 0.001^a^^,^^†^	< 0.001^d^^,^^*^	< 0.001^d^^,^^*^	< 0.001^d^^,^^*^
Shintoism	14 (3.0)		0 (0.0)		0 (0.0)					
Christianity	17 (3.6)		89 (44.3)		24 (18.6)					
Islam	1 (0.2)		5 (2.5)		20 (15.5)					
Hinduism	0 (0.0)		3 (1.5)		4 (3.1)					
None	342 (73.2)		94 (46.8)		58 (45.0)					
Sleeping hours, median (IQR)	6.5 (6.0–7.0)	482	7.0 (6.5–8.0)	216	7.0 (6.5–8.0)	134	< 0.001^b^^,^^†^	< 0.001^c^^,^^*^	< 0.001^c^^,^^*^	0.576^c^
Alcohol drinking (Yes) (%)	349 (72.0)	485	162 (73.6)	220	75 (55.1)	136	< 0.001^a^^,^^†^	0.644^a^	< 0.001^a^^,^^†^	< 0.001^a^^,^^†^
Having chronic conditions need regular checkups (%) (Yes)	87 (17.9)	485	41 (18.6)	220	13 (9.6)	136	0.048^a^^,^^†^	0.824^a^	0.019^a^	0.020^a^
Medical-related license (%) (Yes)	379 (81.7)	464	167 (97.1)	172	85 (68.0)	125	< 0.001^a^^,^^†^	< 0.001^a^^,^^†^	< 0.001^a^^,^^†^	< 0.001^a^^,^^†^
Living with somebody (Yes) (%)	313 (67.0)	467	192 (89.3)	215	47 (37.3)	126	< 0.001^a^^,^^†^	< 0.001^a^^,^^†^	< 0.001^a^^,^^†^	< 0.001^a^^,^^†^
Current work status (full-time, part-time) (Yes) (%)	364 (80.9)	450	90 (53.9)	167	21 (16.5)	127	< 0.001^a^^,^^†^	< 0.001^a^^,^^†^	< 0.001^a^^,^^†^	< 0.001^a^^,^^†^
Work experience, Median (IQR)	7.0 (3.0–12.0)	381	3.0 (1.0–8.0)	105	2.0 (0.0–5.0)	94	< 0.001^b^^,^^†^	< 0.001^c^^,^^*^	< 0.001^c^^,^^*^	0.027^c^

*n* = the total number of answers for each question.

a chi-square test,

b Kruskal-Wallis test,

c Mann-Whitney *U*-test,

d Fisher exact test.

† Statistically significant at 0.05,

* Statistically significant at the 0.017 level after Bonferroni correction.

### Comparison of preventive health behavior, perceived control, and perceived health competence in three student groups

Preventive behaviors were significantly different between Japan (Median = 41.0, IQR = 37.0–45.0) and the United States (Median = 37.0, IQR = 33.0–41.0), including international students, respectively (Z = −8.699, *P* < 0.001). Among the three student groups (Table 2), Japanese students had the highest preventive behavior total score compared to the American students (Z = −9.448, *P* < 0.001) and international students (both countries) (Z = −2.779, *P* = 0.005). Contrarily, Japanese students had the lowest perceived control compared to the American students (Z = −9.797, *P* < 0.001) and international students (both countries) (Z = −5.220, *P* < 0.001). Japanese students had lower perceived health competence than the American students (Z = −5.868, *P* < 0.001); however, they did not show significant differences with international students at an alpha level of 0.017. Between the American and international students, the American students had higher perceived health competence (Z = −2.691, *P* = 0.007), but they did not show any significant differences in perceived control.

**Table 2:** Comparison of preventive behavior, perceived control, perceived health competence among three student groups.

	**Japan** **(*N* = 485)** **group 1**	**USA** **(*N* = 220)** **group 2**	**International students in** **Japan and the U.S** **(*N* = 136)** **group 3**	***P*** **-value**			
	**Median (IQR)**	**Median (IQR)**	**Median (IQR)**	**All groups**	**1 vs. 2**	**1 vs. 3**	**2 vs. 3**
Preventive behaviors	42 (38.0–45.0)	37 (33.0–40.0)	40 (35.0–44.0)	< 0.001^a^^,^^†^	< 0.001^b^^,^^*^	0.005^b^^,^^*^	< 0.001^b^^,^^*^
Perceived control	31 (28.0–35.0)	35 (32.0–38.0)	35 (30.0–37.0)	< 0.001^a^^,^^†^	< 0.001^b^^,^^*^	< 0.001^b^^,^^*^	0.022^b^
Perceived health competence	28 (23.0–32.0)	31.5 (27.0–33.0)	29 (25.0–32.8)	< 0.001^a^^,^^†^	< 0.001^b^^,^^*^	0.022^b^	0.007^b^^,^^*^

† Statistically significant at 0.05,

* Statistically significant at 0.017 level after Bonferroni correction,

a Kruskal-Wallis test,

b Mann-Whitney *U*-test.

The median scores of each item included in all three scales among three student groups are summarized in (Supplementary Tables 1, 4, 5).

### Factors related to preventive behavior

Table 3 summarizes the factors related to preventive behavior in bivariate analysis among three student groups. Sex, alcohol drinking, living with someone, current work status, sleeping hours, perceived control, and perceived health competence were significantly related to the preventive behavior, at least in one group, and were entered into multivariate binary logistic regression. Living condition, sleeping hours, and perceive health competence showed opposite association with preventive behaviors among different student groups (Table 3). Therefore, these factors combined with student groups as interaction terms were also entered into logistic regression. Scatter plot indicated all three student groups had positive association between perceived control and preventive health behaviors, but the line was the highest for the Japanese students and the lowest for the American students (Supplementary Figure 1). Similar results were obtained when the two outliers (Japanese student = 1 and American student = 1) were removed (Supplementary Figure 2).

**Table 3:** Factors related to preventive behavior in bivariate analysis.

**Mann-Whitney *U*-test**		**Japanese students (*****N*** = **485)**		**American students (*****N*** = **220)**		**International students (*****N*** = **136)**	
		**Mean rank**	***P*-value**	**Mean rank**	***P*-value**	**Mean rank**	***P*-value**
Sex	Male	213.5	0.003^*^	90.4	0.032^*^	60.0	0.090^*^
	Female	252.5		112.7		71.2	
Alcohol drinking	Yes	234.0	0.023^*^	109.5	0.696	66.0	0.411
	No	266.1		113.3		71.6	
Living with someone^†^	Yes	235.9	0.659	105.1	0.044^*^	66.1	0.536
	No	230.1		132.7		62.0	
Have any license	Yes	238.1	0.057	87.17	0.206	61.8	0.795
	No	207.5		58.80		63.6	
Working currently	Yes	230.4	0.079	91.2	0.038^*^	68.6	0.535
	No	203.4		75.6		63.1	
Have religious beliefs^†^	Yes	226.7	0.477	104.7	0.330	60.0	0.091
	No	236.7		96.8		71.2	
Chronic conditions^†^	Yes	257.9	0.274	126.0	0.085	52.2	0.201
	No	239.8		107.0		69.9	
**Spearman correlation**		ρ	***P*** **-value**	ρ	***P*** **-value**	ρ	***P*** **-value**
Age		0.055	0.236	0.111	0.104	−0.003	0.970
Sleeping hours		0.019	0.683	0.138	0.043^*^	−0.031	0.726
Work experience		0.060	0.242	0.004	0.970	0.018	0.863
Perceived control		0.169	< 0.001^*^	0.221	< 0.001^*^	0.173	0.044^*^
Perceived health competence		0.171	< 0.001^*^	−0.113	0.096	−0.012	0.888

* Significant level at 0.05. Each category high Mean rank indicating better preventive behavior.

† Variables showed opposite association with preventive behavior among student groups.

In multivariate logistic regression, compared to Japanese students, American students (adjusted odds ratio: AOR = 0.149, 95% confidence interval: CI = 0.091–0.244, *P* < 0.001) had lower preventive behavior (Table 4). Higher perceived control (AOR = 1.055, CI: 1.021–1.089, *P* < 0.001) was significantly related to better preventive behavior. In addition, female students were nearly twice as likely to have better preventive behavior than male students in both countries. We entered interaction terms such as “student groups^*^living conditions,” “student groups^*^sleeping hours,” and “student groups^*^perceived health competence” in logistic regression analysis. However, due to student groups^*^sleeping hours (VIF = 51.648), student groups^*^perceived health competence (VIF = 37.620) showed high multicollinearity, and only “student groups^*^living conditions” was kept in the final model. Perceived health competence was not associated with preventive behavior.

**Table 4:** Factors related to preventive behaviors among different student groups.

	**B**	**AOR (95% CI)**	***P*-value**	**VIF**
**Japanese students as a reference**				
American students	−1.904	0.149 (0.091–0.244)	< 0.001^*^	3.113
International students	−0.314	0.730 (0.350–1.525)	0.403	2.109
Perceived control	0.053	1.055 (1.021–1.089)	0.001^*^	1.233
Perceived health competence	0.016	1.016 (0.989–1.045)	0.252	1.141
Female	0.616	1.852 (1.304–2.629)	< 0.001^*^	1.062
Alcohol drinking	−0.299	0.742 (0.518–1.062)	0.102	1.033
Living with somebody	0.260	1.297 (0.833–2.021)	0.250	1.649
Currently working	0.354	1.424 (0.958–2.116)	0.080	1.408
Sleeping hours	0.012	1.012 (0.868–1.179)	0.879	1.139
**Japanese students × living alone as a reference**				
American students × living with somebody	0.705	2.025 (0.584–7.022)	0.266	1.211
International students × living with somebody	−0.345	0.708 (0.285–1.759)	0.457	3.218

Multivariate binary logistic regression: Nagelkerke R^2^ = 0.180, Hosmer and Lemeshow test (χ^2^ = 11.358, P = 0.182). Durbin-Watson value = 1.963, AOR, adjusted odds ratio; CI, confidence interval; VIF, variance inflation factor. Preventive behavior was sorted into two categories from the median scores (≥44.0 or < 44.0).

* Significant level at 0.05.

In the second logistic regression with five nationality categories, American students (AOR = 0.133, CI: 0.080–0.233, *P* < 0.001) had lower preventive behaviors as compared to the Japanese students (Table 5). Higher perceived control and being female were also associated with better preventive behavior in this model. Perceived health competence was not associated with preventive behavior.

**Table 5:** Factors related to preventive behaviors among different nationalities.

	**B**	**AOR (95%CI)**	***P*-value**	**VIF**
**Japan as a reference**				
Mainland China	−0.526	0.591 (0.204–1.709)	0.332	2.512
The United States	−2.016	0.133 (0.080–0.223)	< 0.001^*^	1.545
Other Asian countries or regions	−0.715	0.489 (0.206–1.163)	0.106	2.526
Non-Asian countries	−0.015	0.985 (0.181–5.364)	0.986	3.184
Perceived control	0.064	1.066 (1.031–1.103)	< 0.001^*^	1.259
Perceived health competence	0.020	1.020 (0.991–1.049)	0.173	1.147
Female	0.618	1.855 (1.299–2.649)	< 0.001^*^	1.054
Alcohol Drinking	−0.345	0.708 (0.491–1.022)	0.065	1.033
Living with somebody	0.278	1.321 (0.843–2.070)	0.225	1.649
Currently working	0.337	1.401 (0.940–2.086)	0.098	1.387
Sleeping hours	−0.001	0.999 (0.855–1.166)	0.985	1.143
**Japan × living alone as a reference**				
China Mainland × living with somebody	1.190	3.288 (0.740–14.613)	0.118	2.586
The United States × living with somebody	0.555	1.741 (0.479–6.334)	0.400	1.204
Other Asian countries or regions × living with somebody	−0.497	0.608 (0.195–1.895)	0.391	2.588
Non-Asian countries × living with somebody	−1.464	0.231 (0.030–1.763)	0.158	3.289

Multivariate binary logistic regression: Nagelkerke R^2^ = 0.205. Hosmer and Lmeshow test (χ^2^ = 4.124, P = 0.846). Durbin-Watson value = 1.983. AOR, adjusted odds ratio; 95% CI, 95% confidence interval; VIF, variance inflation factor. Preventive behavior was sorted into two categories from the median scores (≥44.0 or < 44.0).

* Significant level at 0.05.

### Factors related to perceived control

Factors related to perceived control in bivariate analysis among each student groups were entered in multivariate binary logistic regression (see Supplementary Table 6). In logistic regression, result indicated that compared to Japanese students, American students showed nearly four times higher perceived control after controlling for sex, religious beliefs, work experience, and interaction terms “student groups^*^have religious beliefs (or not)” and “student groups^*^work experience” (Table 6). There was no significant association between religion or interaction of religion and student groups and perceived control.

**Table 6:** Factors related to perceived control among different student groups.

	**B**	**AOR (95%CI)**	***P*-value**	**VIF**
**Japanese students as a reference**				
American students	1.349	3.854 (1.723–8.619)	0.001^*^	2.982
International students	0.499	1.648 (0.763–3.558)	0.204	2.853
Have religious beliefs	0.516	1.675 (0.665–4.222)	0.274	1.837
Work experience	−0.013	0.987 (0.959–1.016)	0.369	1.376
**Japanese students × not have religious beliefs as a reference**				
American students × have religious beliefs	−0.241	0.786 (0.278–2.217)	0.649	2.752
International students × have religious beliefs	0.021	1.021 (0.281–3.712)	0.974	3.234
**Japanese students × lower work experience as a reference**				
American students × higher work experience	−0.013	0.987 (0.917–1.062)	0.719	1.891
International students × higher work experience	0.059	1.061 (0.904–1.245)	0.466	2.12

Multivariate binary logistic regression: Nagelkerke R^2^ = 0.125. Hosmer and Lemeshow test (χ^2^ = 1.483, P = 0.993). Durbin-Watson value = 2.145, AOR, adjusted odds ratio; 95% CI, 95% confidence interval; VIF, variance inflation factor. Perceived control was sorted into two categories from the median scores (≥33.0 or < 33.0).

* Significant level at 0.05.

In the analysis among different nationalities, compared to Japanese students, American students showed 4.5 times higher perceived control (Table 7). No other factors were significant.

**Table 7:** Factors related to perceived control among different nationalities.

	**B**	**AOR (95%CI)**	***P*-value**	**VIF**
**Japan as a reference**				
Mainland China	0.019	1.02 (0.397–2.620)	0.968	1.805
The United States	1.506	4.51 (1.952–10.423)	< 0.001^*^	2.925
Other Asian countries or regions	0.745	2.105 (0.505–8.770)	0.306	5.512
Non-Asian countries	0.532	1.703 (0.198–14.619)	0.628	3.891
Have religious beliefs	0.29	1.337 (0.833–2.146)	0.229	1.841
Work experience	−0.014	0.986 (0.958–1.014)	0.326	1.377
**Japan × lower work experience as a reference**				
China Mainland × higher work experience	0.200	1.221 (0.860–1.743)	0.264	1.562
The United States × higher work experience	−0.022	0.978 (0.909–1.053)	0.555	2.696
Other Asian countries or regions × higher work experience	0.035	1.035 (0.842–1.273)	0.743	5.223
Non-Asian countries × higher work experience	−0.054	0.947 (0.630–1.424)	0.795	3.912
**Japan × not have religious beliefs as a reference**				
China Mainland × have religious beliefs	−0.608	0.544 (0.100–2.968)	0.482	1.63
The United States × have religious beliefs	0.017	1.018 (0.356–2.907)	0.974	1.855
Other Asian countries or regions × have religious beliefs	0.341	1.406 (0.297–6.658)	0.667	2.54
Non-Asian countries × have religious beliefs	1.265	3.543 (0.219–57.292)	0.373	2.343

Multivariate binary logistic regression: Nagelkerke R^2^ = 0.135. Hosmer and Lemeshow test (χ^2^ = 1.072, P = 0.998). Durbin-Watson value = 2.179. AOR, adjusted odds ratio; 95% CI, 95% confidence interval; VIF, variance inflation factor. Perceived control was sorted into two categories from the median scores (≥33.0 or < 33.0).

* Significant level at 0.05.

### Factors related to the perceived health competence

Factors related to perceived health competence in bivariate analysis among each student group are summarized in the Supplementary Table 7. In multivariate binary logistic regression, American students showed nearly three times higher perceived health competence than Japanese students (AOR = 2.840, CI: 1.430–5.643, *P* = 0.003) (Table 8). However, American students with a chronic illness (AOR = 0.084, CI: 0.022–0.314, *P* < 0.001) had lower perceived health competence than Japanese students without a chronic illness. In addition, higher perceived control (AOR = 1.088, CI: 1.048–1.129, *P* < 0.001) was significantly related to higher perceived health competence. Other factors did not show any significant association with perceived health competence.

**Table 8:** Factors related to perceived health competence among different student groups.

	**B**	**AOR (95%CI)**	***P*-value**	**VIF**
**Japanese students as a reference**				
American students	1.044	2.840 (1.430–5.643)	0.003^*^	1.672
International students	0.478	1.613 (0.820–3.173)	0.166	1.833
Female	−0.145	0.865 (0.574–1.303)	0.487	1.095
Perceived control	0.084	1.088 (1.048–1.129)	< 0.001^*^	1.152
Have any license	0.733	2.082 (0.767–5.655)	0.150	1.152
Currently working	0.351	1.420 (0.808–2.495)	0.223	1.642
have chronic condition	−0.068	0.934 (0.530–1.646)	0.813	1.536
sleeping hours	0.119	1.126 (0.945–1.342)	0.186	1.119
Work experience	0.025	1.025 (0.996–1.055)	0.092	1.163
**Japanese students × not have chronic conditions as a reference**				
American students × have chronic conditions	−2.48	0.084 (0.022–0.314)	< 0.001^*^	1.815
International students × have chronic conditions	−1.299	0.273 (0.043–1.713)	0.166	1.246

Multivariate binary logistic regression: Nagelkerke R^2^ = 0.168. Hosmer and Lemeshow test (χ^2^ = 12.843, P = 0.117). Durbin-Watson value = 1.999. AOR, adjusted odds ratio; 95% CI, 95% confidence interval; VIF, variance inflation factor. Perceived health competence was sorted into two categories from the median scores (≥29.0 or < 29.0).

* Significant level at 0.05.

### Factors related to preventive behavior using SEM

**Figure 2:**
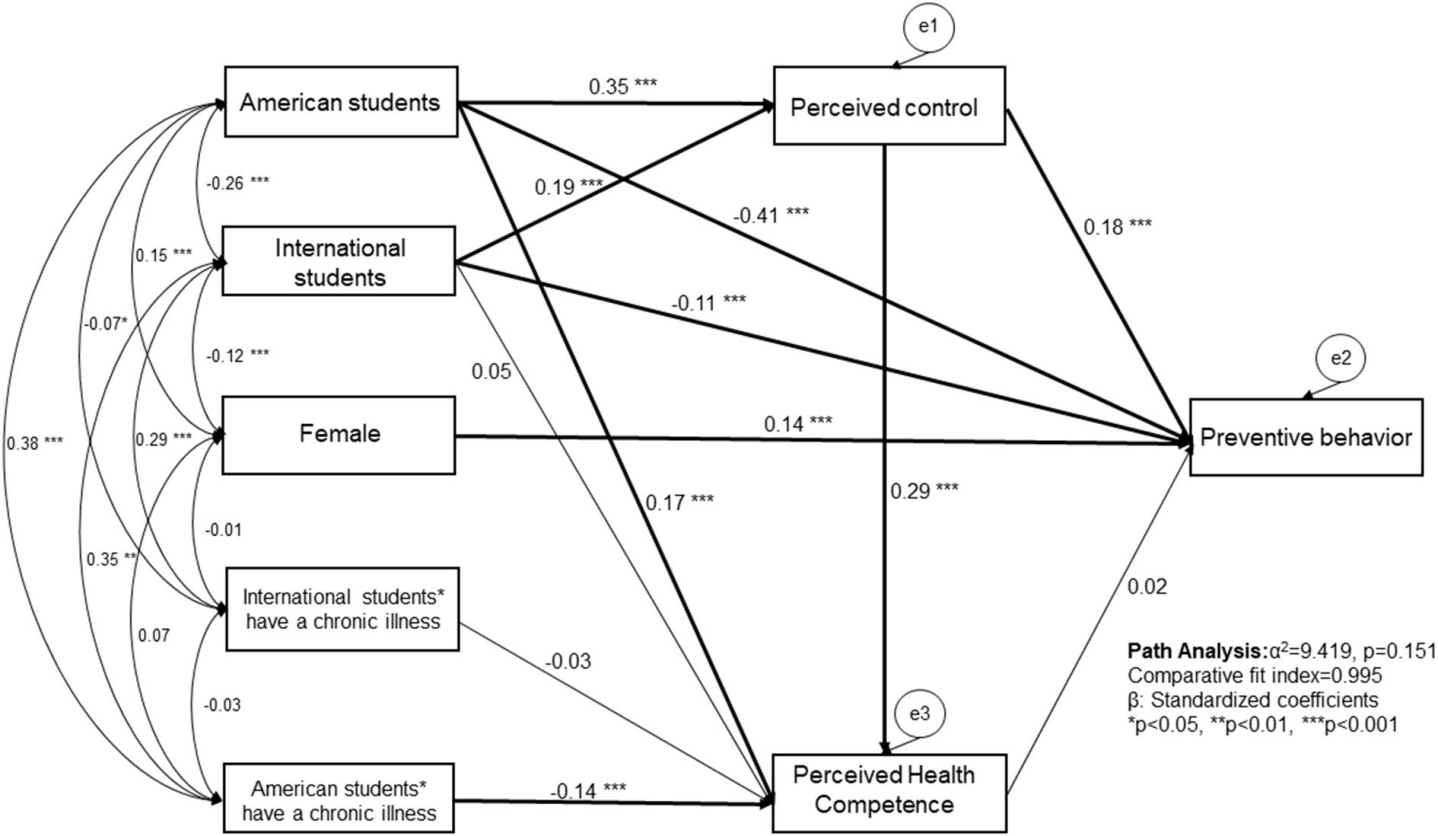
Factors related to perceived control and preventative health behaviors in Japanese, American, and international healthcare graduate students under the COVID-19 pandemic.

Figure 2 displayed the association among variables under SEM with three different student groups. Model fit was good (χ^2^ = 9.419, *P* = 0.151, comparative fit index = 0.995, RMSEA = 0.026). Compared to Japanese students, American students (β = −0.407, *P* < 0.001) and international students (β = −0.112, *P* < 0.001) had significantly lower preventive behavior. Higher perceived control (β = 0.175, *P* < 0.001), and being female (β = 0.141, *P* < 0.001) were related to better preventive behavior. Perceived health competence was not associated with preventive behavior (β = 0.025, *P* = 0.470). When perceived control was removed from the model, perceived health competence showed a positive correlation with preventive behavior (β = 0.007, *P* = 0.027).

In this model, American (β = 0.346, *P* < 0.001) and international students (β = 0.188, *P* < 0.001) had significantly higher perceived control compared to Japanese students. Higher perceived control (β = 0.295, *P* < 0.001) was related to higher perceived health competence. While American students, in general, had significantly higher perceived health competence (β = 0.170, *P* < 0.001) compared with Japanese students, American students who had chronic illness (β = −0.143, *P* < 0.001) had lower perceived health competence compared with Japanese students without chronic illness. The variable “have religious beliefs or not” was not included in the final model because the variable was not significantly related to perceived control or preventive behaviors, and model fit got worse (χ^2^ = 30.736, *P* < 0.001, comparative fit index = 0.969, RMSEA = 0.054) when the variable was added.

**Figure 3:**
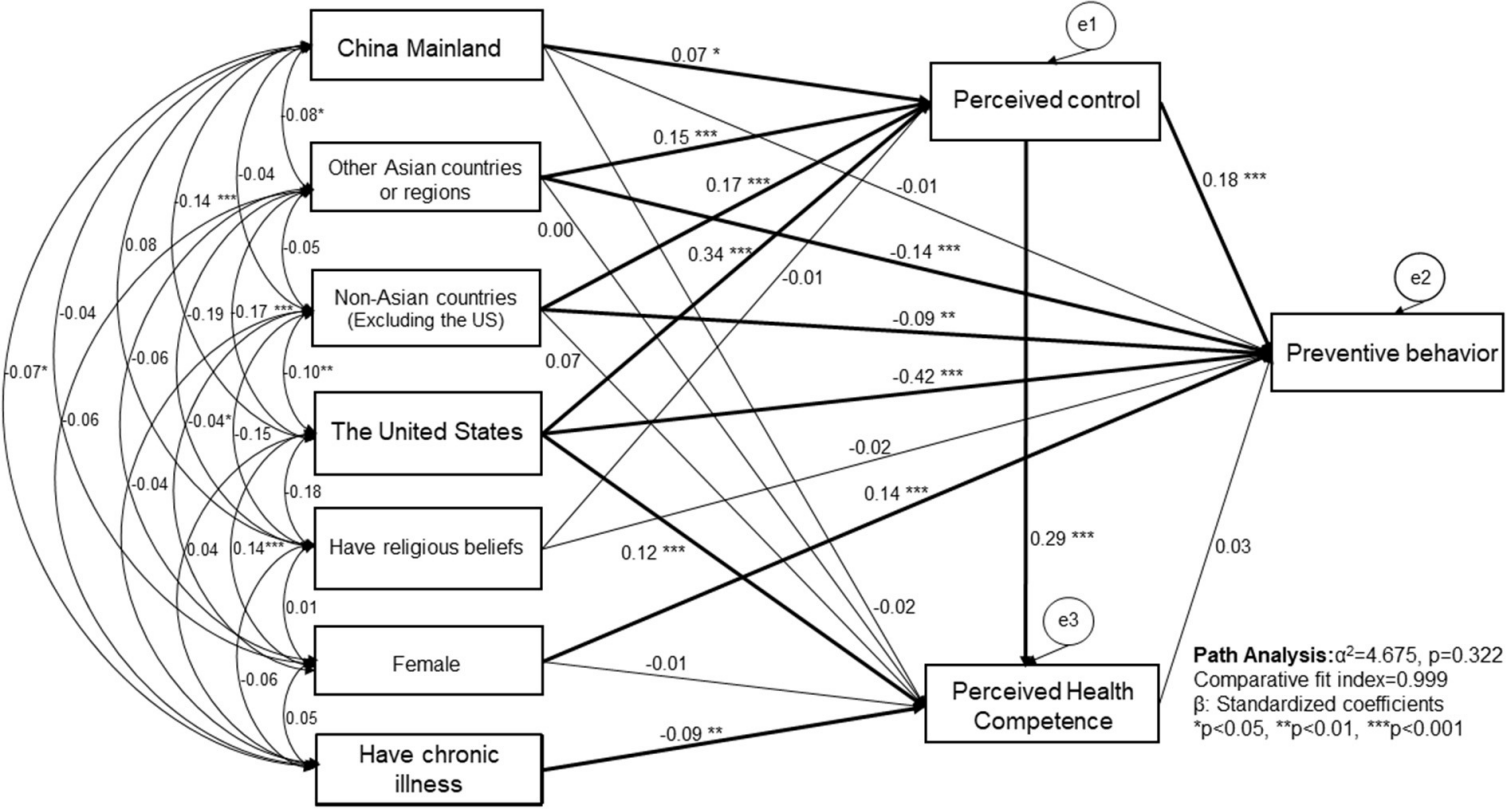
Factors related to perceived control and preventative health behaviors in healthcare graduate students among different nationalities under the COVID-19 pandemic.

Figure 3 reported the association among variables in SEM with five different nationality categories. Compared to Japanese students, American students (β = −0.416, *P* < 0.001), students from other Asian countries or regions (β = −0.136, *P* < 0.001) and non-Asian countries (β = −0.095, *P* = 0.005) had lower preventive behavior. In addition, higher perceived control (β = 0.183, *P* < 0.001) and being female (β = 0.138, *P* < 0.001) were associated with better preventive behavior. Compared to Japanese students, all the other student groups showed significantly higher perceived control. Compared to Japanese students, only American students showed higher perceived health competence (β = 0.122, *P* < 0.001). In addition, factors related to higher perceived health competence were higher perceived control (β = 0.293, *P* < 0.001) and not having chronic conditions (β = −0.093, *P* = 0.004). Religion was not associated with either perceived control or preventive behavior. Model fit was found to be good (χ^2^ =4.675, *P* = 0.322, comparative fit index = 0.999, RMSEA = 0.014).

## Discussion

In this study, although Japanese students showed the lowest perceived control, they had better preventive behavior than the other two student groups of American and international students in both countries. Overall, perceived control was a major predictor of preventive behavior, in addition to student groups and sex. Nationality was not only the strongest predictor of perceived control, which could affect preventive behavior, but could also affect preventive behavior directly.

Hypothesis 1: Levels of perceived control and preventive health behavior differ among three student groups; Japanese students will have the lowest perceived control.

Hypothesis 1 was entirely confirmed. Japanese students' perceived control was the lowest. This is in agreement with the result of Hornsey et al. study (25). Preventive behavior differed among student groups, with the Japanese students showing significantly higher preventive behavior than the other student groups. Previous studies on COVID-19 preventive behavior argued that the differences in preventive behavior between countries might result from the differences in restrictions and recommendations in these countries (15, 51). However, our findings showed differences in preventive behavior between domestic and international student groups in the same country, where the restriction and recommendation conditions were the same. This may have resulted from the difference in information inquiry sources or usual health behavior habits between two student groups. It was shown that international students have tendency to seek information from their local sources (52).

Hypothesis 2: Perceived control, nationality, and religion are associated with preventive behavior.

Hypothesis 2 was partly confirmed in that perceived control was associated with preventive behaviors, but religion was not. More policy level or external perceived control was positively associated with better preventive behavior, which is also consistent with previous studies on individual level perceived control (23, 24, 29, 30). People who believe that they have influence over policy decisions in their communities and strong control over their own behavior, may be more actively engaged in preventive behavior to avoid getting infected. Although positive association was confirmed between perceived control and preventive behavior among all student groups, when nationality is concerned, Japanese students with the lowest perceived control showed higher preventive behavior than other groups. These results indicate that nationality is a stronger predictor of preventive health behavior than perceived control, as also indicated in odds ratios. Actually, previous studies reported weaker association between perceived control and health outcomes in Japanese elderly people than American elderly people (53, 54), an outcome that was consistent with young adults in this study. This can be one reason why Japanese students showed the lowest perceived control but the highest preventive behaviors.

There should be many potential factors included in the variable “nationality.” Differences in pandemic severity, COVID-19 related regulations or recommendations, and vaccine availability in different countries during the study period may influence preventive behavior (55, 56). Stringency indexes of governments' non-pharmaceutical interventions during the pandemic based on nine indicators (such as school closures and restrictions in movement etc.) showed, the indexes of Japan, the United States, and China during the study period of January to May 2021 were 48.15–49.07, 71.76–56.02, and 78.24–55.20, respectively (57). Although the stringency index in the United States and China were on a decline, Japan showed lowest stringency throughout the study period. Even so, Japan's preventive behavior engagement was higher than the other two countries in our findings. China has adopted stricter restriction measures than other countries (58), which may have impacted their acceptance to the social-level preventive measures.

In our study period, the severity of the pandemic was much higher in the US than in Japan, with 7-day average new cases of around 2,00,000 in the early January 2021 which decreased to around 20,000 in late May 2021. During the same period, the numbers in Japan started with ~3,500 cases, and increased to 5,000 (59). However, all the states in the U.S had opened vaccine eligibility to residents aged 16 and over, while in Japan it was only available for those 65 years or older and front-line workers. It is possible that the lack of access to vaccines for non-frontline students in Japan impacted their high engagement in preventive behavior (60, 61).

Another reason why nationality had greater association with preventive behavior than perceived control could be that cultural characteristics (individualism or collectivism) may directly and more strongly influence preventive behavior than mediating effect through perceived control. Collectivistic cultures advocate the obligation to maintain collective harmony, while individualistic cultures promote individual freedom and uniqueness, and people in these cultures act and react based on their own ideas (62). Most Asian cultures tend to be collectivistic (63). A higher preventive behavior among students from Asian countries, including Japan, may have resulted from their higher sense of obligation to maintain collective harmony. Therefore, they may have higher tendency to follow guidelines from authorities on prevention. It is noteworthy, however, that students from non-Asian countries (excluding USA) in this study included only 26 students. Interpretation needs caution, and the results encourage further study with larger representative sample to confirm the association between the nationalities and preventive behavior.

Moreover, usual health habits may also play a role in high preventive behavior. Prior to the current pandemic, Japanese culture or customs already included a habit of social distancing and wearing masks when they had common infectious diseases (e.g., influenza). Such habits also could have helped ensure a smooth transition to preventive behavior engagement during the COVID-19 pandemic (64). As one of the limitations of the current study, usual health habit dimension of participants was not included in the measurement; therefore, direct influence of such cultural/custom dimension to preventive behavior needs further investigation in future studies.

Religious community activity has been shown to be associated with individual health behaviors such as eating habits, physical activity, and smoking (65, 66). However, religion was not associated with infection preventive behaviors in this study. A study reported that churches have limited or totally suspended their religious life in community-based dimension to prevent infection though they maintained contact with the believers in different ways, using modern technologies and access to public media (67). This study included students with different religions with different sample size. Therefore, association between “having religion” and preventive behavior may not be evident or attenuated.

In this study, being female was also associated with better preventive behavior among healthcare graduate students. This result was consistent with previous studies that women tend to be more cautious and preventive against infectious diseases (37, 68).

Perceived health competence was positively correlated with health behavior and health-promoting lifestyle in previous studies among college students (69, 70). However, the current study did not show correlation between perceived health competence and preventive behavior. Positive correlation was observed when perceived control variable was removed from the SEM. Stronger correlation between perceived control and preventive behavior may have resulted in weakened association between perceived health competence and preventive behavior when both variables were entered into the SEM. These results indicate that policy level perceived control was more strongly associated with preventive health behavior than individual health control, compared with previous studies that mainly measured individual-level perceived control (23, 24, 28–30). It is necessary to confirm if this is a pandemic-specific phenomenon.

Hypothesis 3: Nationality and religion are the main predictors of the level of perceived control during the pandemic.

Hypothesis 3 was partly confirmed in that nationality predicted level of perceived control, but religion did not. Students from the United States had the highest perceived control, followed by the non-Asian countries (excluding the US), other Asian countries or regions (excluding Japan and China), and Mainland China. Japanese students showed the lowest perceived control. In a previous study which examined the desire of control and work place choices in people with different cultures showed that Japanese people had less desire for control (i.e., the motivation to have control over various events) than North Americans and Germans, which reflected their tendency to choose workplaces which emphasize belonging and bonding with employees, while the latter groups put greater emphasis on individual achievement (71).

In addition, Hornsey et al. (25) suggested that the influence of Buddhist philosophy in the Japanese culture may play a role in their lowest perceived control among 38 nations, because compared to other western philosophies and traditions, Buddhism advocates that happiness comes when people stop trying to control or change the world and accept its natural flow. Although majority of Japanese participants in our study identified as non-religious, the social culture or tradition they follow in daily life may be greatly influenced by the Buddhist philosophy. It is possible that such a social culture has an effect on an individuals' perceived control even though the individual identifies as non-religious (72, 73). Similarly, participants from China, another country greatly influenced by Buddhism, had similar level of perceived control as the Japanese. However, this study only used the variable “have religious beliefs or not,” which was not found to be significantly related to perceived control. It is necessary to further investigate association between different religious beliefs and perceived control with a larger sample size.

Similar to preventive behavior, perceived control among students from different countries may also be influenced by different cultural orientations, such as individualistic or collectivistic. In individualistic cultures, people are dominated by “self,” with the individual self-forms the primary concept of selfhood, and assertiveness and competitiveness are prioritized (27). Therefore, students from individualistic cultural nations, such as the United States, may think that the individual or “self” has more influence on the surroundings and is more able to control or change it.

In the current study, age; other sociodemographic factors, such as having medical-related license (or not), living with somebody or not; and lifestyle and health-related factors, did not show an association with perceived control or preventive behavior. This may be because the participants in this study were all graduate healthcare students with relatively similar education levels and socio-economic status.

### Limitation

Many limitations are identified in this study. First, this study employed a cross-sectional design, in which causal interpretations between variables are impossible. Second, our samples come from two universities in Tokyo, Japan, and one university in Seattle, USA; therefore, participants and infection status would not be representative of each country. Third, sample size of international students, especially in the United States, was small; therefore, it was combined with international students in Japan. Due to the small sample size, it was not possible to identify differences in international students between the two countries. In our study, there was one Japanese international student in the US and no American international students in Japan. Meanwhile, the students from other Asian and non-Asian countries or regions were also analyzed as a category; therefore, their heterogeneity also could have affected the results of combined groups. In addition, it should be noted that international students who participated in our study were living in a foreign country and therefore may not be representative of the students in their native country. While SEM indicated significant association between nationality, perceived control, and preventive behaviors, participants may not be a representative sample of each location. Therefore, this study indicates the necessity for future research with larger samples and random sampling procedures to confirm the associations. Fourth, in this study, nationality was identified as a major factor associated with perceived control and preventive behavior. Difference in cultural orientation was speculated as the factor that results in differences among nationalities. However, cultural variables, such as collectivism or individualism, of participants were not measured. In addition, some keywords such as “neighborhood,” “vote,” and “party” in items 1, 2, and 7 of the CASE scales may have been different among countries, which could have affected participants' answers. Future research needs to include more specific cultural dimensions, including religious customs and political systems in each country, to obtain a more comprehensive view of culture and policy's role in perceived control and health behaviors. Logistic regressions indicated that only around 20% of the variance related to preventive behavior were explained in this study. There should be more potential factors, such as other health habits or customs, that can be related to preventive behaviors, which need to be explored in future studies.

## Conclusion

The Japanese students had the lowest perceived control followed by international students but showed the highest preventive behaviors, whereas the American students displayed the lowest preventive behavior. Nationality was identified as the main factor associated with both perceived control and preventive behavior. Higher perceived control and being female were also associated with better preventive behavior. Policy level perceived control was more strongly associated with preventive health behavior than individual level perceived health competence.

## Data availability statement

The raw data supporting the conclusions of this article will be made available by the authors, without undue reservation.

## Ethics statement

The studies involving human participants were reviewed and approved by Medical Research Ethics Committee at the Tokyo Medical and Dental University (Approval number: M2020-171), University of Washington Human Subjects Division (HSD) (Approval number: TUDY00012203), and St. Luke's International University Research Ethics Review Committee (Approval number: 20-A096). The patients/participants provided their written informed consent to participate in this study.

## Author contributions

RA: methodology, formal analysis, investigation, data curation, and writing—original draft. AK: conceptualization, methodology, investigation, data curation, funding acquisition, and writing—review and editing. EO: investigation. KN: methodology and investigation. All authors critically reviewed and approved the final manuscript.

## Funding

This study is conducted by the Scientific Research Fund; Japan Society for the Promotion of Science (19K10794). The principal investigator was AK.

## Conflict of interest

The authors declare that the research was conducted in the absence of any commercial or financial relationships that could be construed as a potential conflict of interest.

## Publisher's note

All claims expressed in this article are solely those of the authors and do not necessarily represent those of their affiliated organizations, or those of the publisher, the editors and the reviewers. Any product that may be evaluated in this article, or claim that may be made by its manufacturer, is not guaranteed or endorsed by the publisher.
