# Supplementary figures and images for: Healthcare graduate students' perceived control and preventive behavior for COVID-19 in Japan and the United States: A cross-sectional study
Source: Front Public Health. 2022 Oct 27;10:965897. doi: 10.3389/fpubh.2022.965897 (PMC9648134; doi:10.3389/fpubh.2022.965897)

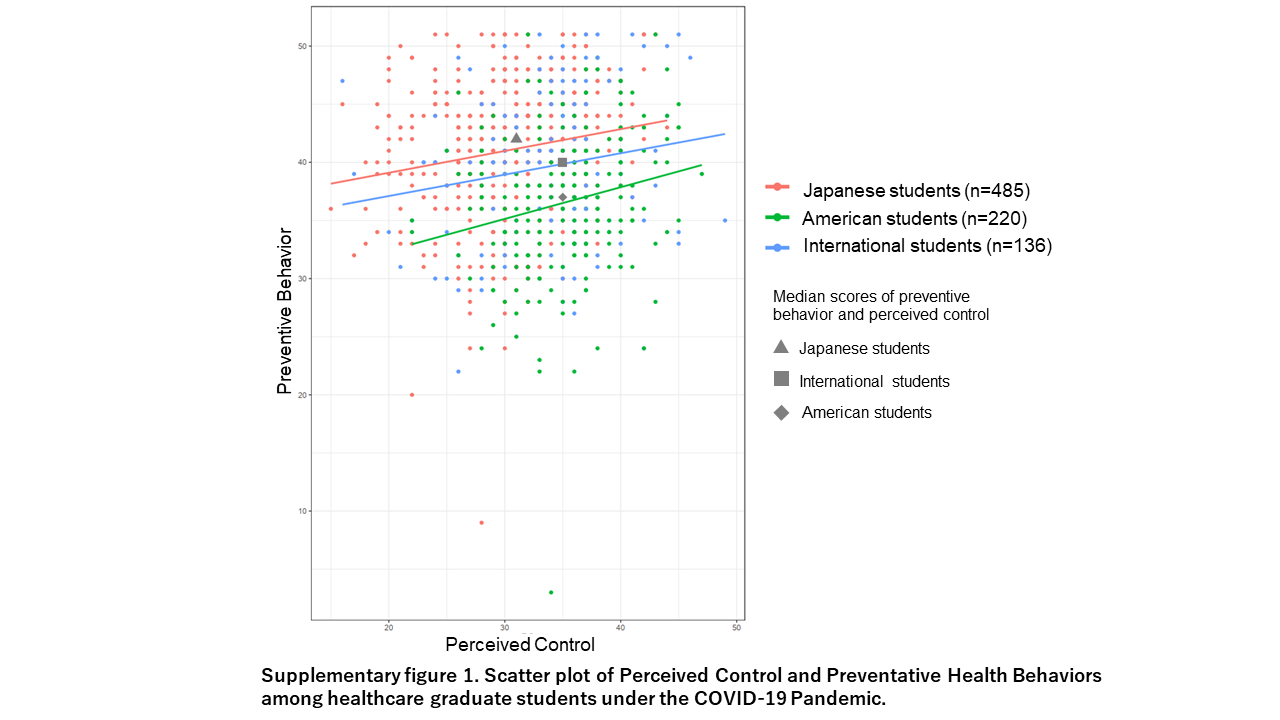

Supplement: Supplementary file 1 [file Image_1.TIF]

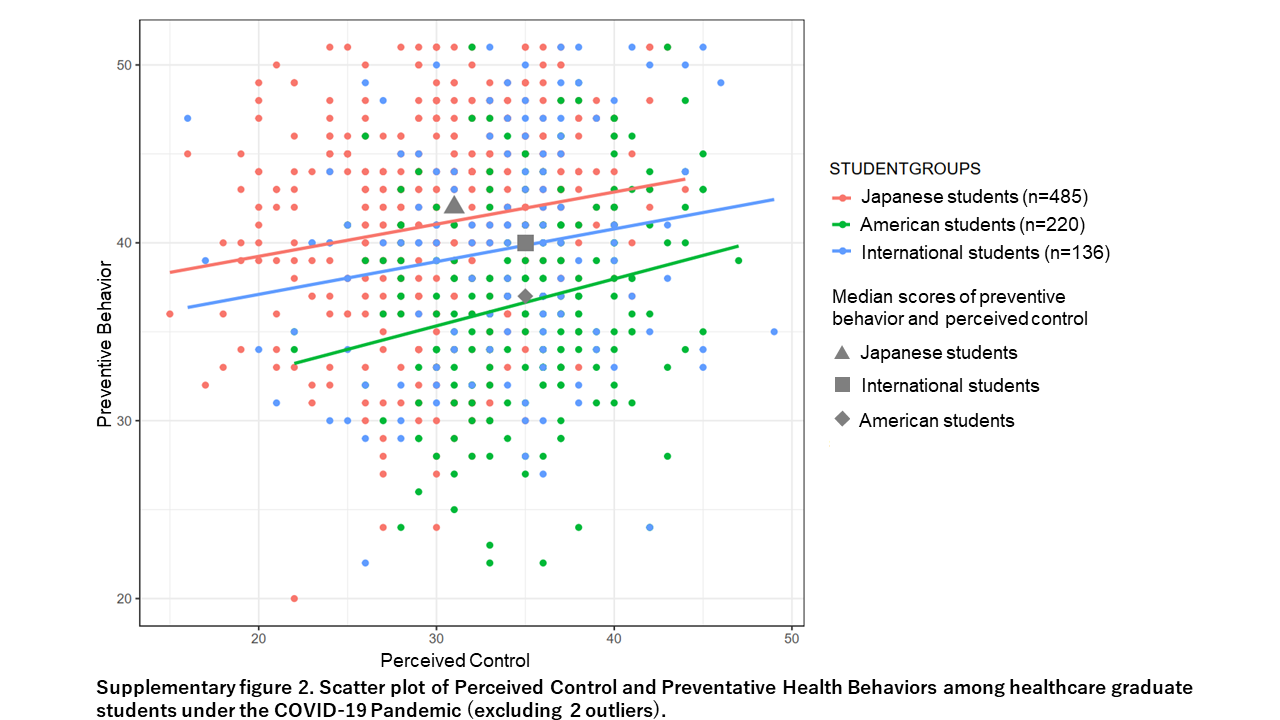

Supplement: Supplementary file 2 [file Image_2.TIF]
