# Supplementary material for: Healthcare graduate students' perceived control and preventive behavior for COVID-19 in Japan and the United States: A cross-sectional study
Source: Front Public Health. 2022 Oct 27;10:965897. doi: 10.3389/fpubh.2022.965897 (PMC9648134; doi:10.3389/fpubh.2022.965897)
Supplement: Supplementary file 3 [file Table_1.DOCX]

**Supplementary table 1. Comparison of preventive behaviors median scores among three student groups**

|  | Japanese students (N=485) | American students  (N=220) | International students in both countries  (N=136) | p value ^a^ |
| --- | --- | --- | --- | --- |
|  | Median (IQR) | Median (IQR) | Median (IQR) |  |
| 1. Wear a mask in public. | 3.0 (3.0-3.0) | 3.0 (3.0-3.0) | 3.0 (3.0-3.0) | <0.001 |
| 2. Self-monitor for respiratory symptoms (cough, runny nose, fever, sore throat, or dyspnea). | 3.0 (3.0-3.0) | 3.0 (3.0-3.0) | 3.0 (3.0-3.0) | 0.361 |
| 3. Follow respiratory hygiene recommendations (eg, covering coughs or sneezes by wearing a face mask or handkerchief, and washing hands often). | 3.0 (3.0-3.0) | 3.0 (3.0-3.0) | 3.0 (3.0-3.0) | 0.001 |
| 4. Avoid hand to face (especially, eyes, mouth, and nose) contact. | 3.0 (2.0-3.0) | 2.0 (1.0-3.0) | 2.0 (2.0-3.0) | <0.001 |
| 5. Check body temperature regularly. | 3.0 (1.0-3.0) | 1.0 (0.0-2.0) | 1.0 (1.0-2.0) | <0.001 |
| 6. Wash hands or use hand sanitizers after touching objects and surfaces in public. | 3.0 (2.0-3.0) | 3.0 (2.0-3.0) | 3.0 (2.0-3.0) | <0.001 |
| 7. Clean and disinfect shared objects and surfaces. | 3.0 (2.0-3.0) | 2.0 (1.0-3.0) | 2.0 (2.0-3.0) | 0.089 |
| 8. Clean and disinfect packaged products. | 1.0 (0.0-2.0) | 1.0 (0.0-1.0) | 2.0 (1.0-2.0) | <0.001 |
| 9. Gargle immediately coming back home. | 3.0 (2.0-3.0) | 0.0 (0.0-0.0) | 1.0 (0.0-2.0) | <0.001 |
| 10. Limit international or domestic travel only to the essential. | 3.0 (3.0-3.0) | 3.0 (2.0-3.0) | 3.0 (3.0-3.0) | <0.001 |
| 11. Limit usage of public transportation only to the essential. | 3.0 (1.0-3.0) | 3.0 (3.0-3.0) | 3.0 (2.0-3.0) | <0.001 |
| 12. Avoid crowded, closed, and close-contact settings. | 3.0 (2.0-3.0) | 2.0 (2.0-3.0) | 3.0 (2.0-3.0) | <0.001 |
| 13. Limit gatherings only to the essential. | 3.0 (2.0-3.0) | 2.0 (2.0-3.0) | 3.0 (2.0-3.0) | <0.001 |
| 14. Maintain social distance (two meters) in public areas. | 3.0 (2.0-3.0) | 2.0 (2.0-3.0) | 2.0 (2.0-3.0) | 0.038 |
| 15. Avoid contact with individuals at high risk for severe illnesses (e.g. elders). | 3.0 (2.0-3.0) | 3.0 (2.0-3.0) | 3.0 (3.0-3.0) | 0.001 |
| 16. Keep up with the latest information and recommendation from health authorities. | 3.0 (2.0-3.0) | 2.0 (2.0-3.0) | 3.0 (2.0-3.0) | 0.004 |
| 17. Follow government physical/social distancing (stay home) orders/requests. | 3.0 (2.0-3.0) | 3.0 (2.0-3.0) | 3.0 (2.0-3.0) | 0.235 |

Note: a: Kruskal-Wallis test. IQR: interquartile range. The significant level at 0.05.

| Comparison of preventive behavior, perceived control, perceived health competence among 4 student groups | | | | | | | | | Pairwise comparison between groups (p value) ^a^ | | | | | | |
| --- | --- | --- | --- | --- | --- | --- | --- | --- | --- | --- | --- | --- | --- | --- | --- |
|  | Japan  (N=485)  Group 1 | | International in Japan  (N=125)  Group 2 | | The United States  (N=220)  Group 3 | | International in the USA  (N=11)  Group 4 | | 1 vs. 2 | 1 vs. 3 | 1 vs. 4 | 2 vs. 3 | 2 vs. 4 | 3 vs. 4 |  |
|  | Mean (SD) | Median (IQR) | Mean (SD) | Median (IQR) | Mean (SD) | Median (IQR) | Mean (SD) | Median (IQR) |  |  |  |  |  |  |  |
| Preventive behaviors | 41.1 (5.7) | 42 (38-45) | 39.5 (6.5) | 40 (34.5-44) | 36.6 (6.1) | 37 (33-40) | 40.7 (5.7) | 39 (36-47) | 0.005* | <0.001* | 0.744 | <0.001* | 0.666 | 0.044 |  |
| Perceived control | 30.8 (5.2) | 31 (28-35) | 33.6 (6.1) | 34 (30-37) | 35.2 (4.7) | 35 (32-38) | 34.6 (5.0) | 36 (30-38) | <0.001* | <0.001* | 0.018 | 0.017 | 0.445 | 0.880 |  |
| Perceived health competence | 27.3 (6.6) | 28 (23-32) | 28.8 (5.1) | 29 (25-32.5) | 30.3 (4.7) | 31.5 (27-33) | 29.2 (5.3) | 32 (25-33) | 0.033 | <0.001* | 0.286 | 0.006* | 0.648 | 0.709 |  |

**Supplementary table 2. Comparison of preventive behavior, perceived control, perceived health competence among 4 student groups**

Note: ^a^: Mann-Whitney U test

*: Statistically significant at 0.008 level after Bonferroni correction

**Supplementary table 3. Summary of international students’ nationalities**

|  | International students in Japan  (N= 118) | International students in The United States  (N= 11) |
| --- | --- | --- |
| Japan | - | 1 |
| China Mainland | 46 | 0 |
| The United States | - | - |
| Other Asian countries or regions (Excluding The USA) | Thailand (n=10), Vietnam (n=9), Myanmar (n=5), Taiwan (n=5), Indonesia (n=5), India (n=5), Bangladesh (n=3), South Korea (n=2), Mongolia (n=2), Pakistan (n=1), Laos (n=1), Nepal (n=1), Afghanistan (n=1) | India (n=5), South Korea (n=2), Taiwan (n=2) |
| Non-Asian countries | Ghana (n=8), Tunisia (n=3), Tanzania (n=2), Germany (n=2), Italy (n=1), Mexico (n=1), Malawi (n=1), Brazil (n=1), Peru (n=1), Egypt (n=1), chili (n=1), | Canada (n=1) |

**Supplementary table 4. Comparison of Perceived control and self-efficacy scale (CASE) median scores among three student groups**

|  | Japanese students (N=485) | American students  (N=220) | International students in both countries  (N=136) | p value ^a^ |
| --- | --- | --- | --- | --- |
|  | Median (IQR) | Median (IQR) | Median (IQR) |  |
| 1. Do you feel you can influence decisions that affect your neighborhood? | 3.0 (2.0-3.0) | 3.0 (3.0-4.0) | 3.0 (3.0-4.0) | <0.001 |
| 2. By working together, people in my neighborhood can influence decisions that affect the neighborhood? | 3.0 (2.0-4.0) | 4.0 (3.0-4.0) | 4.0 (3.0-4.0) | <0.001 |
| 3. Do you feel you can influence decisions that affect your local area? | 2.0 (2.0-3.0) | 3.0 (3.0-4.0) | 3.0 (3.0-4.0) | <0.001 |
| 4. By working together, people in my area can influence decisions that affect the local area? | 3.0 (2.0-4.0) | 4.0 (3.0-4.0) | 4.0 (3.0-4.0) | <0.001 |
| 5. People like me have no say in what the government does. * | 4.0 (3.0-4.0) | 3.0 (2.0-4.0) | 2.0 (2.0-3.0) | <0.001 |
| 6. The government generally treats people like me fairly. | 3.0 (2.0-3.0) | 3.0 (3.0-4.0) | 4.0 (3.0-4.0) | <0.001 |
| 7. My vote makes no difference to the outcome of an election. * | 4.0 (3.0-4.0) | 4.0 (3.0-4.0) | 3.0 (2.0-3.0) | <0.001 |
| 8. It really matters which party is in power, because it will affect our lives. | 4.0 (3.0-5.0) | 4.0 (4.0-5.0) | 4.0 (3.0-5.0) | 0.001 |
| 9. I expect things to get better for me in the foreseeable future. | 3.0 (2.0-4.0) | 4.0 (4.0-4.0) | 4.0 (4.0-5.0) | <0.001 |
| 10. I am satisfied with the amount of control I have over decisions that affect my life. | 3.0 (3.0-4.0) | 4.0 (3.0-4.0) | 4.0 (3.0-4.0) | <0.001 |

Note: *: Reversed items, a: Kruskal-Wallis test. IQR: interquartile range. The significant level is at 0.05.

**Supplementary table 5. Comparison of Perceived health and competence scale (PHCS) scales median scores among three student groups**

|  | Japanese students (N=485) | American students  (N=220) | International students in both countries  (N=136) | p value ^a^ |
| --- | --- | --- | --- | --- |
|  | Median (IQR) | Median (IQR) | Median (IQR) |  |
| 1. I handle myself well with respect to my health. | 4.0 (4.0-5.0) | 4.0 (4.0-5.0) | 4.0 (4.0-5.0) | <0.001 |
| 2. No matter how hard I try, my health just doesn’t turn out the way I would like. * | 4.0 (3.0-5.0) | 4.0 (3.0-4.0) | 4.0 (3.0-4.0) | 0.255 |
| 3. It is difficult for me to find effective solutions to the health problems that come my way. * | 4.0 (2.0-4.0) | 4.0 (3.0-4.0) | 4.0 (3.0-4.0) | 0.114 |
| 4. I succeed in the projects I undertake to improve my health. | 4.0 (3.0-4.0) | 4.0 (4.0-4.0) | 4.0 (3.0-4.0) | 0.003 |
| 5. I’m generally able to accomplish my goals with respect to my health. | 4.0 (3.0-4.0) | 4.0 (4.0-4.0) | 4.0 (3.0-4.0) | <0.001 |
| 6. I find my efforts to change things I don’t like about my health are ineffective. * | 3.0 (2.0-4.0) | 4.0 (3.0-4.0) | 3.0 (2.0-4.0) | <0.001 |
| 7. Typically, my plans for my health don’t work out well. * | 3.0 (2.0-4.0) | 4.0 (3.0-4.0) | 4.0 (2.0-4.0) | <0.001 |
| 8. I am able to do things for my health as well as most other people. | 4.0 (3.0-4.0) | 4.0 (4.0-4.0) | 4.0 (3.0-4.0) | 0.004 |

Note: *: Reversed items, a: Kruskal-Wallis test. IQR: interquartile range. The significant level at 0.05.

**Supplementary table 6. Factors related to Perceived control in bivariate analysis**

|  |  | Japanese students  (N=485) |  | American students  (N=220) |  | International students  (N=136) |  |
| --- | --- | --- | --- | --- | --- | --- | --- |
| Mann-Whitney test |  | Mean Rank | P value | Mean Rank | P value | Mean Rank | P value |
| Sex**†** | Male | 231.0 | 0.353 | 119.3 | 0.170 | 68.2 | 0.534 |
|  | Female | 243.3 |  | 105.0 |  | 64.1 |  |
| Alcohol drinking**†** | Yes | 246.4 | 0.393 | 108.8 | 0.499 | 72.0 | 0.245 |
|  | No | 234.3 |  | 115.3 |  | 64.2 |  |
| Living with someone | Yes | 233.7 | 0.949 | 106.0 | 0.170 | 59.3 | 0.317 |
|  | No | 234.6 |  | 124.8 |  | 66.0 |  |
| Have any license | Yes | 233.9 | 0.625 | 87.2 | 0.304 | 63.7 | 0.770 |
|  | No | 226.1 |  | 64.0 |  | 61.6 |  |
| Working currently | Yes | 232.7 | 0566 | 88.0 | 0.241 | 73.1 | 0.215 |
|  | No | 223.8 |  | 79.3 |  | 62.2 |  |
| Have religious beliefs**†** | Yes | 221.89 | 0.240 | 104.73 | 0.330 | 72.32 | 0.014* |
|  | No | 238.43 |  | 96.75 |  | 56.03 |  |
| Chronic conditions**†** | Yes | 243.1 | 0.992 | 95.6 | 0.095 | 61.0 | 0.472 |
|  | No | 243.0 |  | 114.0 |  | 69.3 |  |
| Spearman correlation |  | ρ | P value | ρ | P value | ρ | P value |
| Age**†** |  | -0.031 | 0.504 | 0.094 | 0.169 | 0.071 | 0.419 |
| Sleeping hours**†** |  | 0.069 | 0.133 | -0.009 | 0.897 | 0.117 | 0.177 |
| Work experience**†** |  | -0.037 | 0.477 | -0.003 | 0.997 | 0.211 | 0.042* |

Note: *: Significant level at 0.05. Each category high Mean rank indicating higher perceived control. †: Variables showed an opposite association with Perceived control among student groups.

**Supplementary table 7. Factors related to perceived health competence in bivariate analysis**

|  |  | Japanese students  (N=485) | | American students  (N=220) | | International students  (N=136) | |
| --- | --- | --- | --- | --- | --- | --- | --- |
| Mann-Whitney test |  | Mean Rank | P value | Mean Rank | P value | Mean Rank | P value |
| Sex† | Male | 234.1 | 0.573 | 127.6 | 0.017* | 65.7 | 0.923 |
|  | Female | 241.6 |  | 102.8 |  | 66.3 |  |
| Alcohol drinking | Yes | 245.7 | 0.503 | 111.4 | 0.717 | 71.4 | 0.347 |
|  | No | 236.2 |  | 108.0 |  | 65.0 |  |
| Living with someone† | Yes | 238.2 | 0.333 | 107.5 | 0.725 | 62.6 | 0.838 |
|  | No | 225.4 |  | 112.3 |  | 64.0 |  |
| Have any license† | Yes | 238.9 | 0.029* | 86.3 | 0.790 | 68.0 | 0.023* |
|  | No | 203.9 |  | 92.3 |  | 52.3 |  |
| Working currently† | Yes | 231.4 | 0.046* | 85.5 | 0.657 | 60.0 | 0.580 |
|  | No | 200.4 |  | 82.2 |  | 64.8 |  |
| Have religious beliefs† | Yes | 227.8 | 0.550 | 99.0 | 0.595 | 67.0 | 0.509 |
|  | No | 236.3 |  | 103.3 |  | 62.6 |  |
| Chronic conditions† | Yes | 244.1 | 0.717 | 65.16 | <0.001* | 58.1 | 0.315 |
|  | No | 238.1 |  | 120.9 |  | 69.6 |  |
| Spearman correlation |  | ρ | P value | ρ | P value | ρ | P value |
| Age |  | 0.067 | 0.146 | 0.015 | 0.830 | 0.121 | 0.166 |
| Sleeping hours† |  | 0.139 | 0.002* | -0.089 | 0.193 | 0.097 | 0.263 |
| Work experience |  | 0.080 | 0.120 | 0.075 | 0.448 | 0.242 | 0.019* |
| Perceived control |  | 0.279 | <0.001* | 0.212 | 0.002* | 0.270 | 0.001 |

Note: *: Significant level at 0.05. Each category high Mean rank indicating higher perceived health competence. †: Variables showed an opposite association with perceived health competence among student groups.
