# Supplementary material for: Healthcare graduate students' perceived control and preventive behavior for COVID-19 in Japan and the United States: A cross-sectional study
Source: Front Public Health. 2022 Oct 27;10:965897. doi: 10.3389/fpubh.2022.965897 (PMC9648134; doi:10.3389/fpubh.2022.965897)
Supplement: Supplementary file 4 [file Table_2.DOCX]

**Covid-19 study coding table**

| Column | Variable | Coding | Note |
| --- | --- | --- | --- |
| A-Q | Frequency of preventive health behavior engagement (17items) | Never＝０  Sometimes＝１  Often =2  Always＝3 | The higher the score, the better the health behavior. |
| R-AA | Perceived control and self-efficacy scale (CASE) | Strongly disagree =1  Disagree =2  Uncertain =3  Agree =4  Strongly agree =5 | Items 5,7 were reverse items.  The higher the score, the higher perceived control. |
| AB-AI | PERCEIVED HEALTH COMPETENCE SCALE (PHCS) | Strongly agree =5  Agree =4  Uncertain =3  Disagree =2  Strongly Disagree =1 | Items 2,3,6,7 were reverse items.  The higher the score, the higher perceived health competence. |
| AJ | Countries five categories | Japan=1  Mainland China=2  The United States=3  Other Asian countries or regions=4  Non-Asian countries (Excluding The US) =5 |  |
| AK | Three comparison groups | Japanese students=1  American students=2  International students in both countries=3 |  |
| AL | Four comparison groups | Japanese domestic students=1  International students in Japan=2  US domestic students=3  International students in the US=4 |  |
| AM | Student Status | 0=Not international students  1=International students |  |
| AN | Age | Number filled in tables |  |
| AO | Sex | Male＝１  Female＝２ |  |
| AP | Weight | Number filled in tables (kg) |  |
| AQ | Height | Number filled in tables (cm) |  |
| AR | BMI | Number filled in tables |  |
| AS | Nationality | Japan＝１  Mainland China＝２  Myanmar＝３  Thailand＝４  Vietnam＝５  Ghana＝６  South Korea=8  Bangladesh=9  The United States=10  Taiwan=11  Indonesia=12  India=13  Mongolia=14  Chili=16  Tunisia=17  Germany=18  Italy=19  Mexico=20  Malawi=21  Egypt=22  Brazil=23  Pakistan=24  Peru=25  Laos=26  Nepal=27  Tanzania=28  Afghanistan=29  Canada=30  Philippine=31  Iran=32  Ukraine=33 |  |
| AT | Religious | Buddhism =1  Shintoism＝２  Christianity＝３  Islam＝４  Hinduism＝５  None＝６ |  |
| AU | Have religious beliefs | No =0  Yes=1 |  |
| AV | Currently working | No =0  Yes=1 |  |
| AW | License | Doctors=1  Dentists=2  Nurses=3  Pharmacists=4  Laboratory technicians=5  None=6 |  |
| AX | Have license | No =0  Yes=1 |  |
| AY | Job experience | Number filled in tables |  |
| AZ | Marital status | Single＝１  Married＝２  Partnered  Divorced＝４ |  |
| BA | The school year | The first year＝１  The second year＝２  The third year＝３  The fourth year＝４  The fifth year=5  The sixth year=6  The seventh year=7 |  |
| BB | Living conditions Total | Alone=0  Living with somebody=1 |  |
| BC | Sleeping hours | Number filled in tables |  |
| BD | Alcohol drinking | No=0  Yes=1 |  |
| BE | Smoking | No＝0  Yes＝１ |  |
| BF | Chronic condition Total | No＝0  Yes＝１ |  |
| BG | Frequency of preventive health behavior engagement (17items) total score | Number filled in tables |  |
| BH | Perceived control and self-efficacy scale (CASE) total score | Number filled in tables |  |
| BI | PERCEIVED HEALTH COMPETENCE SCALE (PHCS) total score | Number filled in tables |  |
| BJ | The main source of income | Full time job=1  Part-time job=2  Scholarships=3  Family support=4 |  |
| BK | Annual income | **For Japan:**  Less than 1 million yen/year＝１  1,000,000 ~1,999,999 yen/year＝２  2,000,000 ~ 2,999,999 yen/year＝３  3,000,000 ~ 3,999,999 yen/year＝４  4,000,000 ~ 4,999,999 yen/year＝５  5 million/year or higher＝６  **For The US**  Less than $10,000/Year＝１  $10,000~19,999 /Year＝２  $20,000~29,999 /Year＝３  $30,000~39,999 /Year＝４  $40,000~49,999 /Year＝５  $50,000/Year or higher＝６ |  |
| BL-BM | CASE scale reverse items |  |  |
| BN-BQ | PHCS scale reverse items |  |  |
| BR | Frequency of preventive health behavior engagement (17items) total score (two categories) | Low=1  High=2 |  |
| BS | CASE scale total score (two categories) | Low=1  High=2 |  |
| BT | PHCS scale total score (two categories) | Low=1  High=2 |  |
| BU | American students | Others=0  American students=1 |  |
| BV | International students | Others=0  International students=1 |  |
| BW | Japanese students | Others=0  Japanese students=1 |  |
| BX | Nationality Japan | Others=0  Japanese students=1 |  |
| BY | Nationality Mainland China | Others=0  Chinese students=1 |  |
| BZ | Nationality US | Others=0  American students=1 |  |
| CA | Nationality Other Asian countries or regions | Others=0  Students from other Asian countries or regions =1 |  |
| CB | Nationality Non-Asian countries | Others=0  Students from Non-Asian countries =1 |  |
